# Bronchial asthma control after argon plasma coagulation turbinectomy in patients with chronic rhinitis

**DOI:** 10.1007/s00405-013-2762-z

**Published:** 2013-10-19

**Authors:** Edyta Jura-Szołtys, Rafał Ficek, Joanna Ficek, Jarosław Markowski, Jerzy Chudek

**Affiliations:** 1 ENT Department, Medical University of Silesia, Francuska Str. 20-24, 40-027 Katowice, Poland; 2 Department of Nephrology, Endocrinology and Metabolic Diseases, Medical University of Silesia, Francuska Str. 20-24, 40-027 Katowice, Poland; 3 Department of Pathophysiology, Medical University of Silesia, Medyków Str. 18, 40-752 Katowice, Poland

**Keywords:** Bronchial asthma, Chronic rhinitis, Argon plasma coagulation, Turbinectomy, Asthma control test

## Abstract

Bronchial asthma is frequently accompanied by chronic rhinitis. It has been observed that effective treatment of rhinitis may reduce asthma symptoms. The aim of the study was the evaluation of the control of bronchial asthma symptoms in patients with chronic rhinitis after argon plasma coagulation turbinectomy (APCt). The effect of APCt was assessed in 47 adults with drug-resistant chronic rhinitis and bronchial asthma 3-month post-procedure. Changes of asthma symptoms were scored using Asthma Control Test (ACT). Subjective improvement of nasal congestion 3 months after APCt was observed in 87 % and of rhinorrhoea in 75 % patients. Rhinomanometry showed 219 ± 19 cm³/s increase of flow and 0.75 ± 0.06 Pa/cm³/s reduction of resistance. The prevalence of patients with insufficient bronchial asthma control decreased from 79 to 4 %. The decrease was associated with diminished frequency of eosinophils >20 % in nasal cytology from 83 % pre-procedure to 28 % in the follow-up. The percentage of eosinophils >20 % in cytology before APCt increased the chance for asthma control improvement by 22.8 times. Reduction in symptoms of drug-resistant rhinitis after APCt is followed by significant improvement of asthma control. The most beneficial therapeutic effects of APCt are noted in patients with a high rate of eosinophils in nasal cytology.

## Introduction

Results of epidemiological studies suggest an association between bronchial asthma and chronic rhinitis. The recent concept perceives mucous membrane of upper and lower respiratory tract as one entity, affected by inflammatory process, and maintained by common mechanisms. In favour of this concept is the fact that the morphology of the eosinophilic inflammation of the mucous membrane of the upper and lower respiratory tract is similar, and the pathogenesis of both allergic rhinitis and bronchial asthma is triggered and perpetuated by the same factors, such as: allergens, non-steroidal anti-inflammatory drugs (NSAIDs), infectious agents, as well as environmental pollution [1, 2].

Chronic rhinitis affects over 75 % of patients with atopic asthma and about 80 % of those with non-atopic asthma [3]. Their coexistence deteriorates the clinical course of bronchial asthma. It has been shown that patients with bronchial asthma and symptoms of chronic rhinitis require more intensive pharmacotherapy, usually often report paroxysmal nocturnal dyspnoea, and are much less active. [4]. Signs and symptoms of chronic rhinitis often precede the occurrence of bronchial asthma. Generally, it is considered that effective treatment of chronic rhinitis may prevent the development of asthma, or at least reduce the intensity of bronchial symptoms [1]. Substantial number of patients may, over many years of the disease, develop hypertrophy of the mucous membrane of the inferior nasal turbinates, resistant to pharmacological treatment, manifested by symptoms of continuous nose obstruction and discharge of water-like secretion [5].

Although surgical treatment does not directly affect the course of allergic reaction, the reduction of inferior nasal turbinate hypertrophy may alleviate the symptoms of allergic rhinitis, through the improvement of local flow disturbances [6–8].

In line with the present view, turbinectomy should be carried out employing methods, which enable maintenance of the physiological function of the mucous membrane after the procedure. Mono-polar argon plasma coagulation (APC), performed without contact between the instrument and the tissue, meets the requirements of modern rhino-surgery. The efficiency and safety of this procedure in reducing hypertrophy of the inferior turbinates have been shown in numerous clinical studies [6, 9, 10]. The argon plasma flame produces uniform surface zones of coagulation and devitalization, of the limited depth (maximum 3 mm), followed by interstitial fibrosis of the coagulated tissues [9].

The aim of this study was the assessment of bronchial asthma symptom control in patients with drug-resistant chronic rhinitis, after argon plasma coagulation of the inferior turbinates.

## Materials and methods

Forty-seven patients (23 females and 24 males) in the age range from 23 to 67 years (average age, 49 ± 2 years) with asthma and chronic rhinitis, with symptoms of nasal obstruction caused by hypertrophy of the inferior nasal turbinates diagnosed with first degree of nasal septum deviation in T/S arrangement in rhinoscopy (Table 1); [11], and bronchial asthma were enrolled. These patients were referred to the outpatient ENT clinic by general practitioners and otolaryngologists from other outpatient clinics. Patients with planned conchoplasty were seen by a single otolaryngologist, who qualified them for the procedure, performed it and then followed them in the outpatient clinic. The characteristics of the study group are summarized in Table 2.

**Table 1:** Classification for nasal septum deviation [(Levine and May turbinoseptal deformity (T/S) (1993)] [11]

Grade	Endoscopic view after decongestion
T/S I	Visible lateral and medial surface of the middle turbinate
T/S II	Anterior attachment of the middle turbinate partially covered by a laterally deviated nasal septum
T/S III	Anterior attachment of the middle turbinate completely covered by nasal septum
T/S IV	Nasal septum deviated to the lateral wall of the nasal cavity completely covers the anterior attachment of the middle turbinate

**Table 2:** Characteristics of the study group

Number of patients	47
Female/male	23/24
Average age (years)	49 ± 2 (range 23–67)
Asthma Control Test (ACT)	
<20 pts	37 (79 %)
20–24 pts	10 (21 %)
25 pts	0
Self-reported intensity of nasal symptoms [*N* (%)]	
Nasal stuffiness	
0 = no symptoms	0
1 = mild symptoms	2 (4 %)
2 = moderate symptoms	17 (36 %)
3 = severe symptoms	28 (60 %)
Rhinorrhoea	
0 = no symptoms	0
1 = mild symptoms	12 (26 %)
2 = moderate symptoms	16 (34 %)
3 = severe symptoms	19 (40 %)
Percentage of eosinophils in cytologic swabs from mucous membrane of the inferior nasal turbinates	
>20 %	39 (83 %)
<20 %	8 (17 %)
Total flow (P) [cm³/s]	201 ± 88 (range 69–428)
Total resistance (R) [Pa/cm³/s]	1.39 ± 0.47 (range 0.37–2.13)

(Mean values ± SD)

Patients had persistent symptoms of chronic rhinitis for at least 6 months, resistant to antiallergic medications and intranasal corticosteroids. Self-reported intensity of nasal symptoms (nasal stuffiness and rhinorrhoea) were recorded by patients using a four-point scale (0 = no symptoms, 1 = mild symptoms, 2 = moderate symptoms and 3 = severe symptoms) before and after treatment.

Patients enrolled to the study were controlled by pulmonologists in the outpatient clinic close to the place of residence, often far from the ENT clinic. Asthma Control Test (ACT) recommended by GINA was applied to assess the course of asthma. The therapy for asthma remained unchanged during the study.

Anterior and posterior rhinoscopy, endoscopic examination of nasal cavities and active anterior rhinomanometry in accordance with the recommendations of the Standardisation Committee on Objective Assessment of the Nasal Airway, IRS, and ERS were performed. Following the recommendations, measurements of nasal patency, before and 3 months after conchoplasty, were preceded by a 20-min period of adaptation to laboratory conditions. Throughout the acclimatization time, the patient remained in a sitting position. In each patient, measurements were recorded during five breathing cycles and the mean value was calculated. The values of total flow were analysed and then the total resistance at 150 Pa level was calculated (Rhinotest MP500, Allergopharma, Germany) [12].

Nasal exfoliative cytology samples were obtained by scraping the inferior surface of the inferior turbinate using disposable nasal brushes. Specimens were spread on microscopy slides, fixed in 95 % ethanol and stained with haematoxylin and eosin and Giemsa. Slides were examined using oil immersion light microscopy (1000×). Eosinophil content in swab higher than 20 % was assumed to be characteristic of allergic rhinitis [13, 14].

### Assessment of asthma control

Bronchial asthma control was assessed on the basis of Asthma Control Test (ACT) (Table 3). The ACT questionnaire contains five questions concerning the occurrence of symptoms, use of drugs, impact of the disease upon everyday activities at work and at home, as well as self-assessment of the level of disease control. Each question was provided with a set of five possible answers, grading the complaints. The maximum score was 25 points and was equivalent to very good control of asthma. Poor asthma control was indicated by a score equal to or below 19 points, whereas incomplete control was observed for scores of 20–24 points [15].

**Table 3:** The Asthma Control Test (ACT) [14]

Question	Answers–score (Pts)				
	1	2	3	4	5
1. In the past 4 weeks, how much time did your asthma keep you from getting as much done at work, school or at home?	All of the time	Most of the time	Some of the time	A little of the time	None of the time
2. During the past 4 weeks, how often have you had shortness of breath?	More than once a day	Once a day	3 to 6 times a week	Once or twice a week	Not at all
3. During the past 4 weeks, how often did your asthma symptoms (wheezing, coughing, shortness of breath, chest tightness or pain) wake you up at night or earlier than usual in the morning?	4 or more nights a week	2 or 3 nights a week	Once a week	Once or twice	Not at all
4. During the past 4 weeks, how often have you used your rescue inhaler or nebulizer medication (such as albuterol)?	3 or more times per day	1 or 2 times per day	2 or 3 times per week	Once a week or less	Not at all
5. How would you rate your asthma control during the past 4 weeks?	Not controlled at all	Poorly controlled	Somewhat controlled	Well controlled	Completely controlled

### Argon plasma coagulation turbinectomy (APCt)

**Fig. 1:**
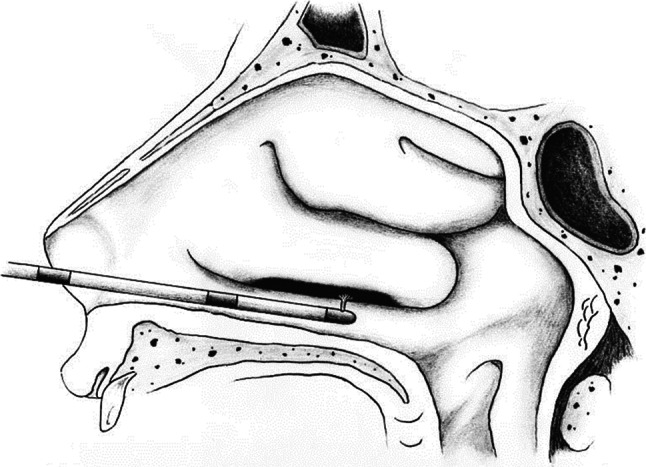
Contactless APC inferior turbinate reduction (APCt)

APCt procedures were performed under local anaesthesia by dressing with 2 % lidocaine solution. Reduction of the lower margins of the inferior nasal turbinate hypertrophy was performed on the entire length of the bottom edge from the rear to the front without contact (Fig. 1), in argon environment with a flow rate of 1.4 l/min and 40 W current power (APC 300 ERBE, ERBE Elektromedizin GmbH, Tuebingen, Germany). The procedure was completely bloodless.

### Follow-up

The initial control visit after APCt was performed after about 7 days post-procedure. In symptomatic patients, the use of intranasal fluticasone furoate was accepted. After a short time, local medication generally was not necessary and was discontinued.

The second control examination was performed after 3 months and included rhinoscopy, endoscopic examination of nasal cavities, nasal cytology, rhinomanometry, ACT and self-reported nasal symptoms questionnaire. Smooth, moist and pale pink mucosa on the lower nasal turbinates was revealed on macroscopic evaluation. Local therapy was not used for at least 3 weeks before reassessment. The study protocol did not interfere with asthma medication.

### Statistical analysis

Statistical analysis was performed using the software package Statistica 8 (StatSoft Polska). Results have been presented as average values ± SEM. In statistical analysis, non-parametric tests were applied: *χ* ^2^ test, *U* Mann–Whitney test, Wilcoxon’s test. Logistic regression analysis was performed for calculation of the chance of improvement of asthma control after APCt. Odds ratio (OR) was presented with 95 % confidence interval. The value of *p* < 0.05 was assumed as the threshold of statistical significance.

## Results

Insufficient (score below 20 points) control of asthma before the procedure was found in 37 patients (79 %) and incomplete control (score of 20–24 points) in 10 patients (21 %). Before the APCt, none of the patients declared full control of bronchial asthma according to ACT test (score of 25 points).

Before APCt, the average values of total flows and resistance were 201 ± 88 cm³/s and 1.39 ± 0.47 Pa/cm³/s, respectively. Prior to the treatment, nasal stuffiness and rhinorrhoea symptoms were evaluated at 2.6 ± 0.6 and 2.1 ± 0.7 pts, respectively (Table 2).

In the study group, in 39 patients (83 %), the nasal cytology revealed the percentage of eosinophils exceeding 20 %.

### Post-procedure follow-up

Immediately after the APCt, a zone of superficial coagulation was noticed in the place of plasma application. Within a week, the coagulated tissue was covered with a fibrin coating, while after 1 month linear scars were observed on the lower margins of the inferior nasal turbinates.

**Fig. 2:**
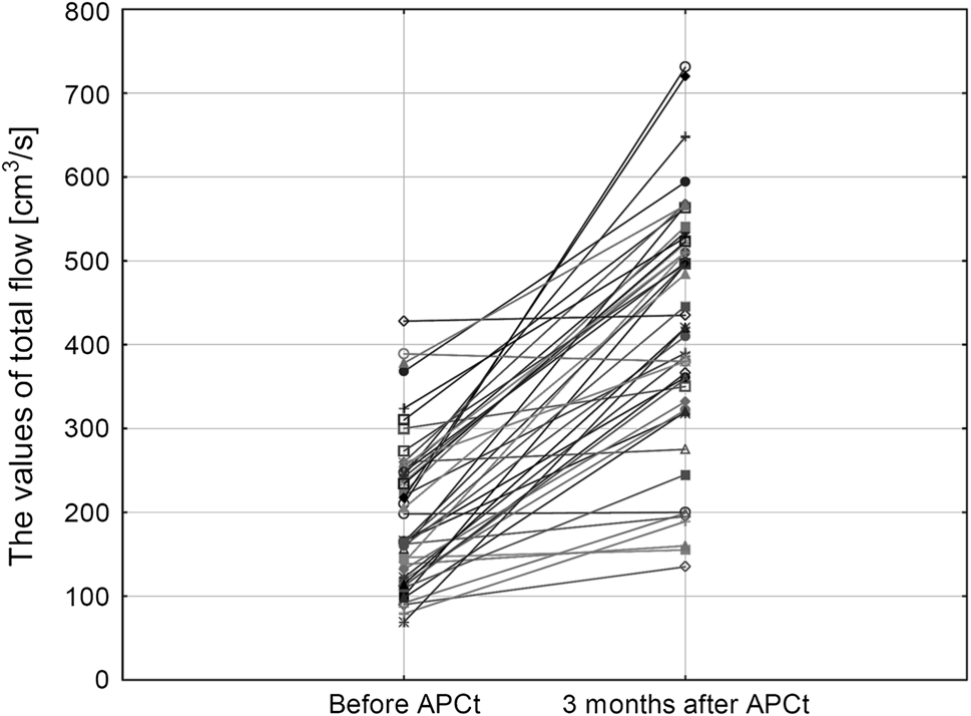
Results of the active anterior rhinomanometry—values of total flow before and 3 months after APCt

**Fig. 3:**
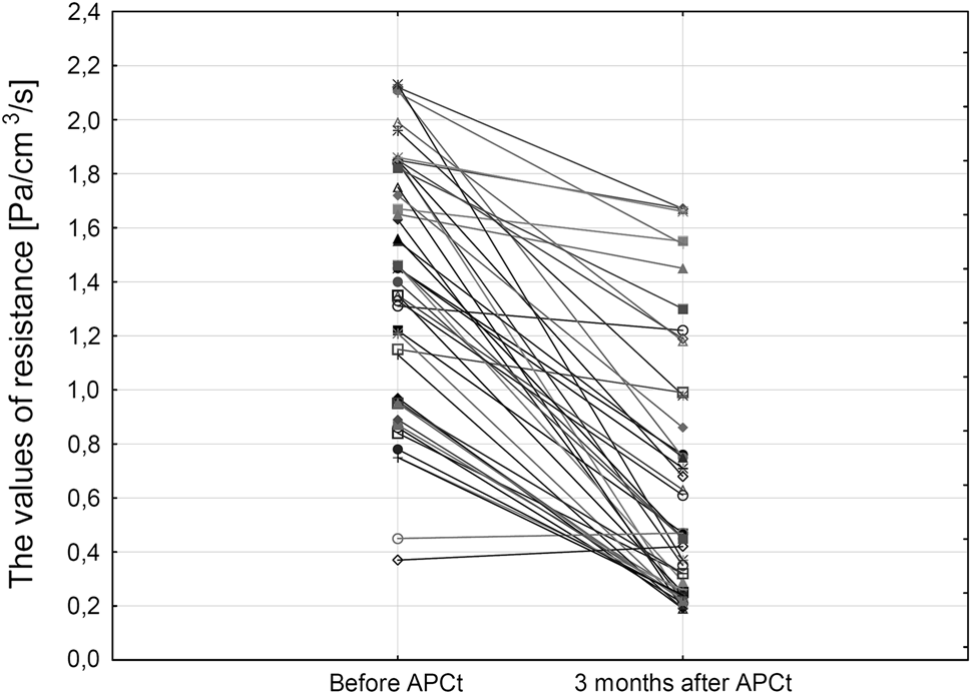
Results of the active anterior rhinomanometry—values of resistance before and 3 months after APCt

Three months after APCt, the average value of total flow increased significantly by 219 ± 19 cm³/s (Fig. 2), whereas the average value of resistance decreased by 0.75 ± 0.06 Pa/cm³/s (Fig. 3). Nasal stuffiness and rhinorrhoea symptoms significantly (*p* < 0.001) improved. The stuffiness score declined from 2.6 ± 0.6 to 1.6 ± 0.7 pts and rhinorrhoea score from 2.1 ± 0.7 to 1.2 ± 0.8 pts post-procedure. The subjective improvement of breathing through the nose was self-reported by 35 out of 47 patients (75 %). Moreover, 34 patients (72 %) reported reduction of the amount of mucoid secretion in nasal cavities and on the posterior wall of the pharynx.

A significant reduction in the number of patients with the percentage of eosinophils in nasal cytology >20 % from 39 (83 %) to 13 (28 %) in the whole group was observed.

In the subgroup of patients with a high percentage of eosinophils in cytology before APCt, remarkably greater increase in total flow (of at least 47 %) in anterior active rhinomanometry was demonstrated (Table 4).

**Table 4:** Results of anterior active rhinomanometry in subgroups of patients with hypertrophy of mucous membrane of the inferior nasal turbinates, qualified for argon plasma coagulation turbinectomy (APCt) according to results of Asthma Control Test (ACT) questionnaire and eosinophilia in cytologic swabs from mucous membrane of the inferior nasal turbinates (more or less 20 %)

	Asthma control test		Eosinophilia	
	<20 pts (*N* = 37)	20–24 pts (*N* = 10)	>20 % (*N* = 39)	≤20 % (*N* = 8)
Total flow (P1) before APCt [cm³/s]	172 ± 65	309 ± 82	190 ± 81	253 ± 112
Total flow (P2) 3 month after APCt [cm³/s]	402 ± 157	489 ± 77	451 ± 135	269 ± 108
Total resistance (R1) before APCt [Pa/cm³/s]	1.5 ± 0.4	1 ± 0.5	1.4 ± 0.4	1.3 ± 0.6
Total resistance (R2) 3 month after APCt [Pa/cm³/s]	0.7 ± 0.5	0.5 ± 0.4	0.5 ± 0.4	1.1 ± 0.5
ΔP [P2–P1]	230 ± 136	180 ± 108	261 ± 102	16 ± 19
ΔR [R2–R1]	−0.8 ± 0.4	−0.5 ± 0.3	−0.9 ± 0.4	−0.2 ± 0.2
ΔP % [100*(P2–P1)/P1]	152 ± 108	69 ± 52	160 ± 95	8 ± 8
ΔR % [100*(R2–R1)/R1]	−56 ± 26	−47 ± 35	−64 ± 19	−8 ± 14

(Mean values ± SD)

### Changes in asthma control

**Fig. 4:**
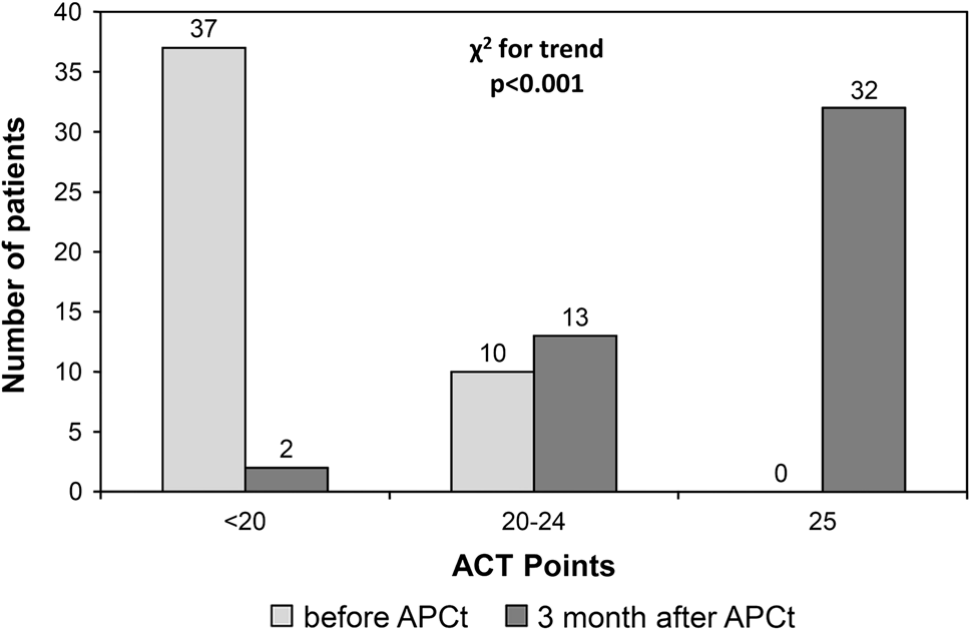
Comparison of results of bronchial Asthma Control Test (ACT) before and 3 months after APCt in 47 patients (*χ*² for trend <0.001)

Three months after APCt, the ACT test revealed full control of asthma (score of 25 points) in 32 patients (68 %), incomplete control (score of 20–24 points) in 13 patients (28 %), and insufficient control only in 2 patients (4 %) (Fig. 4). The improvement in asthma control defined as the change of ACT level was observed in 43 patients (91.5 %).

There was almost twice greater improvement in the total flows in the subgroup of patients with insufficient asthma control before APCt (ACT score below 20 points) than in the subgroup with incomplete asthma control (20–24 points).

Logistic regression analysis showed that the percentage of eosinophils in cytology exceeding 20 % before the procedure significantly increased the chance for the improvement of asthma control level after APCt (OR = 22.8 with 95 % confidence interval of 2.0–263.6; *p* = 0.01).

## Discussion

The obtained results justify a statement that argon plasma coagulation turbinectomy not only ameliorates of the nose patency, due to reduction of hypertrophic mucous membrane of inferior nasal turbinates, but also improves the level of asthma control.

Epidemiological studies indicate that bronchial asthma is one of the most frequent chronic diseases, affecting about 300 million people worldwide [16, 17]. Bronchial asthma, in particular when poorly controlled, negatively influences health-related quality of life, impairing the patient’s everyday functioning, as well as causing a significant economic burden [18, 19].

The currently accepted strategy for treatment of asthma consists of obtaining and maintaining the control of clinical symptoms, minimizing the incidence of aggravations and reducing the number of unscheduled visits to emergency departments and of administered emergency medications, in a way as not to limit the patient’s everyday activity [17].

Chronic rhinitis accompanying bronchial asthma negatively influences its course, while effective management concerning rhinologic complaints results in alleviation of bronchial symptoms [1]. Due to the impairment of the physiological function of the mucous membrane of nasal cavities in the course of chronic rhinitis, the air reaching the bronchi is not sufficiently heated, cleaned, and humidified. For that reason, the bronchial mucous membrane is exposed to irritating factors, which may trigger defence reflex reactions. Moreover, the lack of nasal patency is conducive to fixing a non-physiological route for breathing—through the mouth [2].

In case of ineffective pharmacological treatment in patients with mucous membrane hypertrophy of the inferior nasal turbinates, improvement of the course of rhinitis may be achieved via surgical procedures [19, 20]. In the study, the reduction of hypertrophic mucous membrane of the inferior nasal turbinates was obtained using APCt. Post-procedure, a significant increase of the air flow in rhinomanometry was observed, accompanied by a significant decline in total resistance (Figs. 2, 3). The improvement is comparable to the efficacy of alternative methods of hypertrophic mucous membrane reduction of the inferior nasal turbinates, such as laser or interstitial coagulation [7, 21, 22].

APCt used in our clinic to reduce hypertrophic mucosa of the lower nasal turbinates has many advantages in comparison to other surgical procedures, such as the total or submucosal conchotomy, interstitial electrocautery, cryosurgery or laser surgery.

APCt is one of the best, entirely bloodless methods [9], while total conchotomy is associated with the risk of intra- and post-operative bleeding and may require the use of a front tamponade. The most important advantage of APC conchoplasty is the possibility of performing a non-contact procedure, while cryosurgery or electrocautery require direct contact of the applicator with the hypertrophied mucous membrane [23]. The use of inert gas—argon—during APCt results in lack of smoke formation [9], which in the case of the use of electrocautery or laser may hinder the precise orientation in the operative field [21].

Due to the circumfluent anatomical structures, it is very important for safety that the penetration depth of argon plasma is limited to 3 mm [9]. In contrast to APC, the use of laser causes a thermal reaction in the adjacent tissues [22].

The lack of charring effect of the coagulated tissues during the APCt procedure is very important for the preservation of the physiological function of the nasal mucosa [9]. The effect of tissue charring occurs in the case of laser and cryosurgery usage, causing the formation of deep necrotic lesions.

In addition to the improvement of nasal air flow, reduced amount of mucoid secretion flowing down the posterior wall of the pharynx was found. In accordance with previous reports of other authors, improved nasal patency, as well as reduced volume of secretion, eliminates the negative influence of breathing through the mouth upon the course of bronchial asthma [2, 24].

Of interest, the reduction of symptoms of chronic rhinitis post-procedure was associated with a significant reduction in the prevalence of nasal mucous membrane eosinophilic infiltration. The infiltration is one of the important markers of allergic rhinitis frequently accompanying bronchial asthma [25]. In our study exfoliative cytology was performed to assess the percentage of eosinophils in swabs from the mucous membrane of the inferior nasal turbinates. On the basis of available literature, the percentage of eosinophils over 20 % has been assumed as a value characteristic for allergic rhinitis [13, 26]. Unexpectedly, we found that patients with eosinophilic infiltration of the mucous membrane had markedly greater post-procedure air flow improvement. Each patient with eosinophil percentage exceeding 20 % before APCt achieved at least 47 % post-procedure flow increase. Therefore, our results suggest that nasal cytologic swabs analysis, performed before APCt, may be a new potential prognostic factor for post-procedure flow improvement.

Additionally, after APCt an improvement in the control of bronchial asthma was observed, especially in patients with >20 % eosinophils in nasal cavity swabs before turbinectomy. A high percentage of eosinophils in nasal mucosa infiltrate supposes allergic aetiology of rhinitis and may reflect eosinophilic infiltrations in other parts of the respiratory system, including bronchi [27]. It was shown that eosinophil infiltration of nasal cavities is related both to allergic rhinitis and bronchial asthma [25]. We cannot exclude that the improvement in asthma control observed in our study was secondary to diminished upper airway resistance (nasal stuffiness and rhinorrhoea) and restoration of physiological nasal breathing.

Our results, at least partially, confirm the previous findings showing that difficulties in nasal breathing play an important role in the pathogenesis of asthma exacerbation [28]. In the study, improvement of nasal stuffiness and rhinorrhoea was followed by a significant improvement in asthma control.

Regardless of the association between a high percentage of eosinophils in cytology and its role in the pathogenesis of asthma and allergic rhinitis, its presence before APCt was a significant prognostic factor. The chance for improvement of asthma control was as much as 22.8 times higher when found before APCt. This interesting finding requires confirmation in other prospective studies.

The main limitations of this study are the use of ACT alone for the evaluation of asthma control. However, ACT is considered as a reliable tool for the assessment of asthma control, used for treatment decisions made by asthma specialists, which correlates with other measurements such as spirometry and FeNO [17, 29].

Unfortunately, we do not have data on long-term follow-up. In the majority of patients, the visit after 3 months was the last one in our outpatient clinic.

## Conclusions

(1) Reduction in symptoms of drug-resistant rhinitis after argon plasma coagulation turbinectomy is followed by significant improvement in asthma control. (2) The most beneficial therapeutic effects of argon plasma coagulation turbinectomy are noted in patients with a high rate of eosinophils on nasal cytology. (3) Argon plasma coagulation turbinectomy is a viable method for treating patients with drug-resistant chronic rhinitis and bronchial asthma.
